# *Finback*: a web-based data collection system at SSRF biological macromolecular crystallography beamlines

**DOI:** 10.1107/S1600577523010615

**Published:** 2024-01-19

**Authors:** Feng Yu, Ke Liu, Huan Zhou, Minjun Li, Huating Kong, Kunhao Zhang, Xingya Wang, Weiwei Wang, Qin Xu, Qiangyan Pan, Zhijun Wang, Qisheng Wang

**Affiliations:** a The Division of Life Science, Shanghai Advanced Research Institute, Chinese Academy of Sciences, 239 Zhengheng Road, Pudong, Shanghai 201204, People’s Republic of China; Paul Scherrer Institute, Switzerland

**Keywords:** macromolecular crystallography beamline, data collection, synchrotron beamline control, graphical user interface

## Abstract

An integrated computer software system for macromolecular crystallography (MX) data collection at the BL02U1 and BL10U2 beamlines of the Shanghai Synchrotron Radiation Facility is described. The system, *Finback*, implements a set of features designed for the automated MX beamlines, and is marked with a user-friendly web-based graphical user interface (GUI) for interactive data collection. The *Finback* client GUI can run on modern browsers and has been developed using several modern web technologies including WebSocket, WebGL, WebWorker and WebAssembly. *Finback* supports multiple concurrent sessions, so on-site and remote users can access the beamline simultaneously. *Finback* also cooperates with the deployed experimental data and information management system, the relevant experimental parameters and results are automatically deposited to a database.

## Abstract 2

*Finback* is a biological macromolecular crystallography data collection system that features a user-friendly interactive web-based graphical user interface. The backend is based on the *Experimental Physics and Industrial Control System* and the frontend has been developed with several modern network technologies including WebSocket, WebGL, WebWorker and WebAssembly.

## 1. Introduction

To meet the requirements of highly automatic and reliable operation, a user-friendly and robust data collection system is necessary to integrate beamline hardware and underlying software for biological macromolecular crystallography (MX) beamlines. Since 2010, the *BluIce* system (McPhillips *et al.*, 2002 ▸) has been applied at Shanghai Synchrotron Radiation Facility (SSRF) beamlines. The *BluIce* system was originally developed by Stanford Synchrotron Radiation Lightsource. The SSRF MX group ported *BluIce* and integrated it with a Rigaku ACTOR sample changer for mounting/dismounting samples, remote access and automatic crystal screening. In recent years, with the popularization of hybrid pixel array detectors such as Pilatus and Eiger, the amount of diffraction data collected has increased dramatically. To overcome this challenge, an automatic data processing and experimental information system, which can be cooperated with *BluIce*, was developed and deployed at SSRF MX beamlines (Yu *et al.*, 2019 ▸). However, both SSRF accelerator and optics devices as well as our newly home-made diffractometer and sample changer are controlled using *Experimental Physics and Industrial Control System* (*EPICS*). Therefore, maintenance and development of the distributed hardware servers (DHSs) of *BluIce* requires much extra work.

Besides *BluIce*, over the past 20 years multiple data acquisition software and graphical user interfaces (GUIs) have been developed, including *BSS* (Ueno *et al.*, 2005 ▸), *CBASS* (Skinner *et al.*, 2006 ▸), *STARS* (Yamada *et al.*, 2008 ▸), *MxCuBE2* (Gabadinho *et al.*, 2010 ▸), *JBluIce-EPICS* (Stepanov, Hilgart *et al.*, 2011 ▸; Stepanov, Makarov *et al.*, 2011 ▸; Stepanov *et al.*, 2013 ▸), *GDA* (https://github.com/opengda/gda-diamond) and *DA+* (Wojdyla *et al.*, 2018 ▸). In recent years, to apply the safer and faster-responding remote access mode, several web-based data collection systems have been successfully developed and deployed for many MX beamlines or other beamlines, such as *WIFIP* (Sallaz-Damaz & Ferrer, 2017 ▸), *YAIBEX* (Australian Synchrotron), *MXCuBE3* (Mueller *et al.*, 2017 ▸) and *Daiquiri* (Fisher *et al.*, 2021 ▸). Inspired by these progressions, and further considering that our experimental information system also uses a web interface, developing a data collection system with web interface can integrate the two systems more deeply and reduce the workload by sharing partial source code (such as raw image viewer and database manipulation). Taking these factors into consideration, we have developed *Finback* – a new MX data collection system which implements a set of features expected for an automated MX beamline and includes a user-friendly web-based GUI for interactive data collection. *Finback* is now deployed at SSRF BL02U1 and BL10U2 and has been available to users since June 2021.

## 2. Beamline endstation configuration

Both SSRF BL02U1 and BL10U2 are equipped with a home-made diffractometer and a home-made sample changer called Swordfish. The area detectors are Eiger2 S 9M (BL02U1) and Eiger X 16M (BL10U2), respectively. The data storage server and computing cluster are provided by SSRF Phase II Computing Center, and include about 6.9 PB of storage space and 48 computing nodes (each with two Intel Xeon Gold 6140 CPUs and 128 GB memory) for all SSRF beamlines. The beamline and data storage server are connected via a 40 Gbit network, and the data storage server and the computing nodes are connected via an Infiniband network. For online data processing, an automatic data-processing backend called Seal is deployed that provides a web-based interactive interface known as SealWeb for inspecting the processed results (Yu *et al.*, 2019 ▸). An additional CPU node and a GPU node deployed at each beamline are dedicated to the processing of grid scan X-ray diffraction images and artificial intelligence crystal centering based on Mask-RCNN (He *et al.*, 2017 ▸), respectively. Benefiting from the stream data of the Eiger detector, grid scan images are processed simultaneously during data collection, so real-time grid scan analysis results can be displayed when scanning.

## 3. Software infrastructure

**Figure 1:**
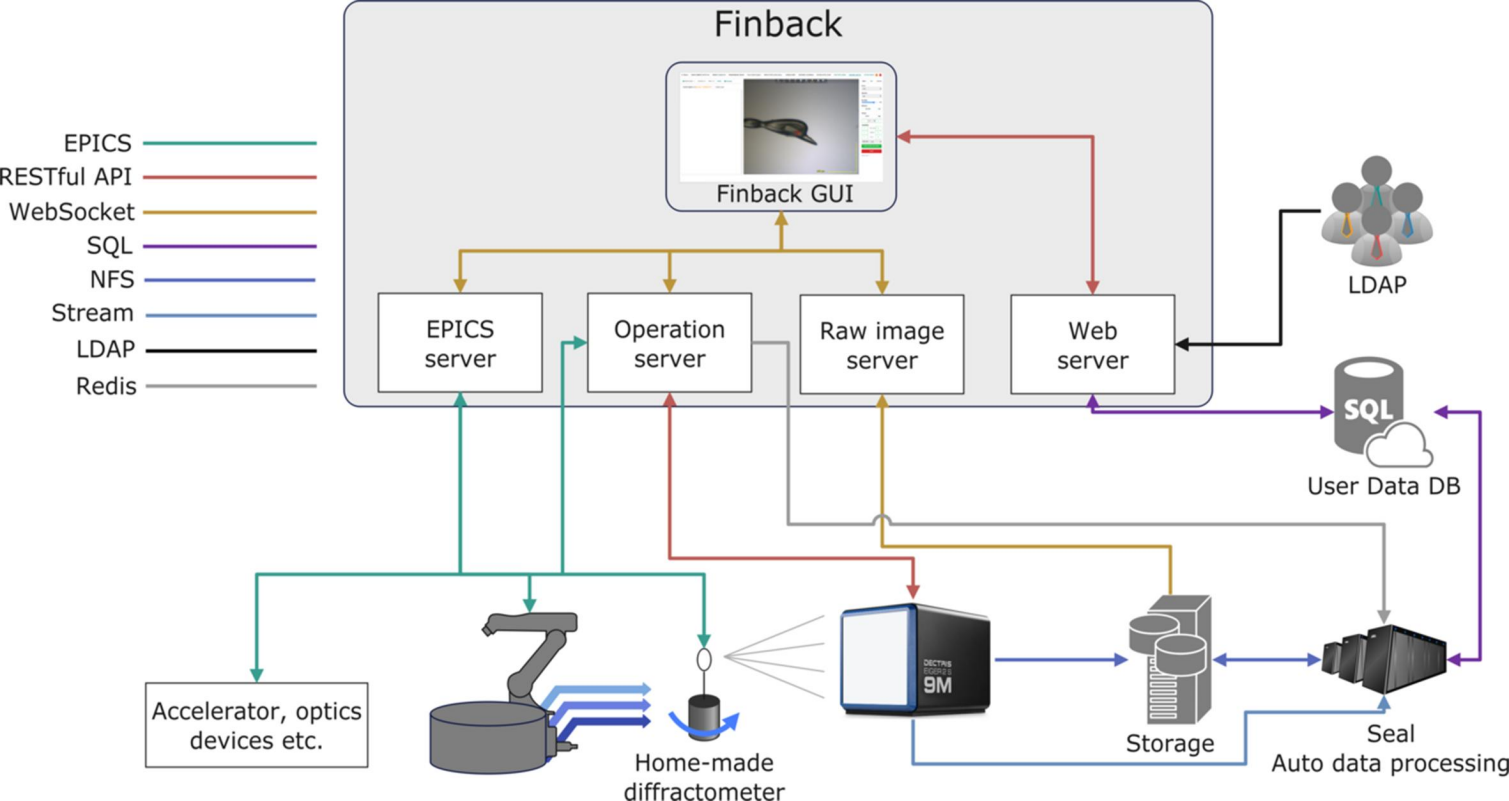
The software infrastructure of *Finback*. The *Finback* components are shown in the shaded box. The communication protocols among the *Finback* components, hardware, LDAP, user data database and automatic processing backend are represented by different colored lines.

The *Finback* data collection system consists of a frontend and backend, as well as a Redis message broker over Transport Layer Security (TLS) and a series of helper scripts for beamline setup, on-site real-time raw image monitoring, *etc*. (Fig. 1 ▸).

The frontend is a web-based interactive interface for user manipulation. The logical code is written based on the *Angular* framework and the GUI is constructed based on the *Fomantic-UI* framework. *Angular*, which is also used in SealWeb, is a popular frontend framework that supports data-driven dynamic rendering of frontend interfaces without page refresh. *Fomantic-UI* is a responsive web design framework that supports dynamic adjustment of page layout according to the actual displayed page size. To reduce maintenance difficulties, the source code of the frontend is strictly identical at both beamlines. Some beamline-specific features can be enabled or disabled by beamline parameters configuration.

Since all devices are controlled using *EPICS*, except for the Eiger detector which is controlled by a RESTful API, it made sense that we chose *EPICS* for the hardware drivers. Therefore, the original DHS drivers are not needed. The *EPICS* system supports asynchronous monitoring of process variable (PV) values, and a call-back can be executed as soon as the value is changed. Using this mechanism, it is possible to monitor a series of specific PVs and transmit the charged values to clients in real time. However, the Http protocol is a half-duplex protocol that cannot support real-time two-way communication between the frontend and the backend. Benefiting from HTML5, the WebSocket protocol has been introduced into modern browsers. Thus, browsers can use sockets to achieve real-time two-way communication between frontend and backend. In *Finback*, WebSocket communication and message parse run as a separate WebWorker thread. The latency of the *Finback* system is similar to other MX data collection systems, such as *BluIce*. In practice, over 600 crystals can be measured in one day at maximum.

The backend consists of the web server, the *EPICS* server, the operation server and the raw image server. The web server is written using *Node.js*, provides login authorization via the light-weight directory access protocol (LDAP), and database operation for sample information and data processing results. The other servers are written using Python, implementing real-time transmission of hardware status, raw diffraction images and user operations.

In the *Finback* client GUI, four different types of commands over WebSocket are supported:

(1) *EPICS* commands. Remote procedure call (RPC) over WebSocket of *caget* and *caput* is partially implemented in the *EPICS* server. *EPICS* commands are mainly used for setting beamline parameters and controlling simple hardware, such as manually switching of the diffractometer shutter.

(2) Device commands. Implemented in the operation server and contain complex hardware movement.

(3) Operation commands. Most frequently used, such as auto-centering and data collection. When an operation command is running, the *Finback* client GUI is locked, and no further command will be executed, except the abort command.

(4) Abort command. Terminates operation commands on demand.

In the *Finback* GUI, there are five operation tabs, namely ‘Collection’, ‘RawImages’, ‘Protocols’, ‘Tools’ and ‘Admin’. Among them, the Collection tab is used for sample exchange and data collection, the RawImages tab is used for raw images inspection, the Protocols tab is used to import sample information, and the Tools and Admin tabs are mainly used by beamline staff.

### 3.1. Collection tab

**Figure 2:**
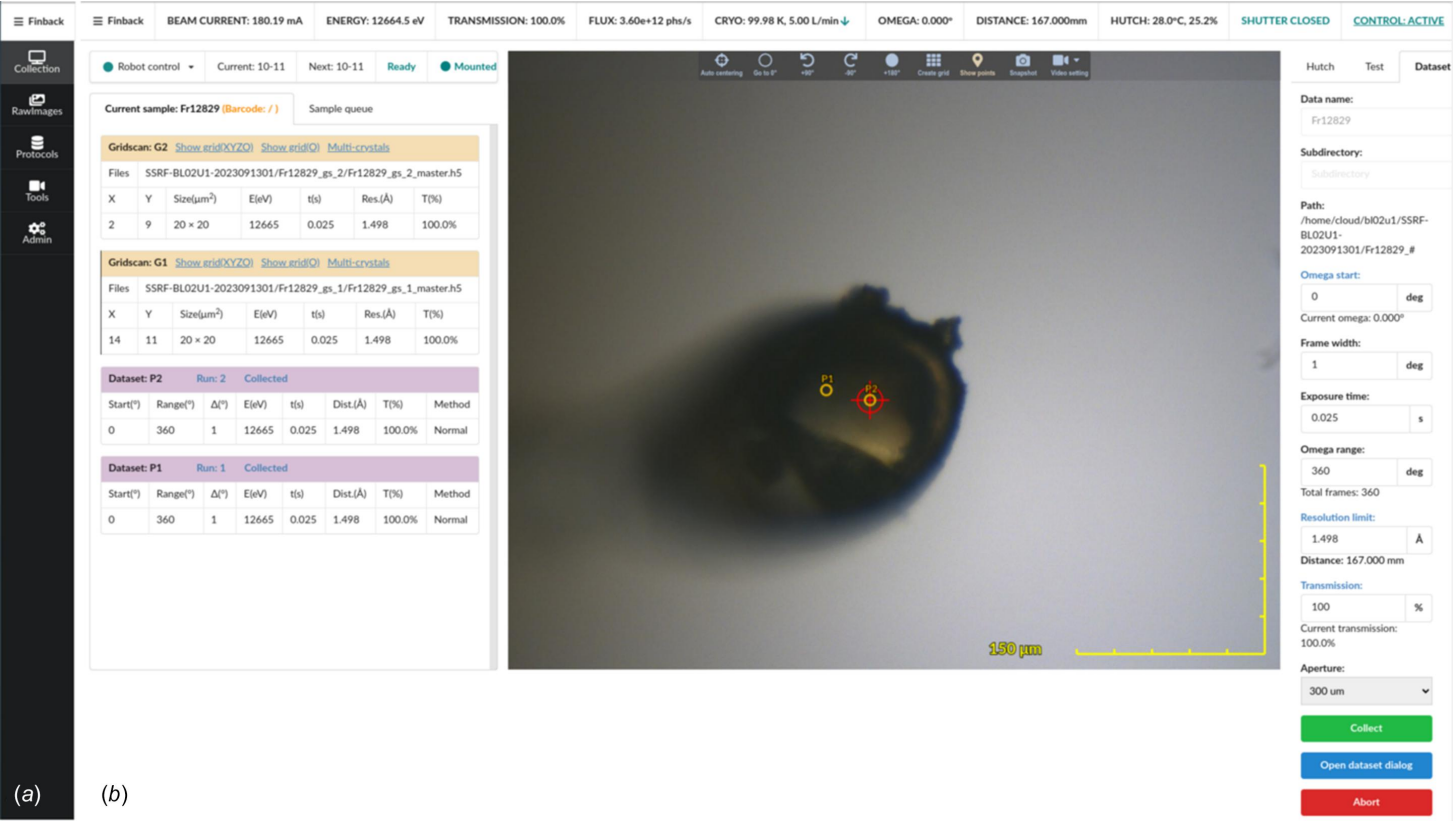
The interface of *Finback*. (*a*) Navigation bar. Five tabs can be chosen. The navigation bar is automatically hidden when it is not active. The ‘Collection’ tab is responsible for sample mount/dismount, fluorescence scanning and data collection. The ‘RawImages’ tab is used to show raw diffraction images. The ‘Protocol’ tab is responsible for importing sample information. The ‘Tools’ tab currently provides real-time video of the experimental station, mainly for remote users to observe the status of the experimental station equipment. The ‘Admin’ tab is used by beamline staff. (*b*) The ‘Collection’ tab. Like *BluIce*, *Finback* supports multiple clients logging in simultaneously, but only one client can operate. The user can switch between active and passive states by clicking the Control button in the upper right corner.

The Collection tab is the most frequently used component in the *Finback* GUI, and includes motor movement, sample exchange, data collection and fluorescence scans, as well as displaying autoindexing results (Fig. 2 ▸).

**Figure 3:**
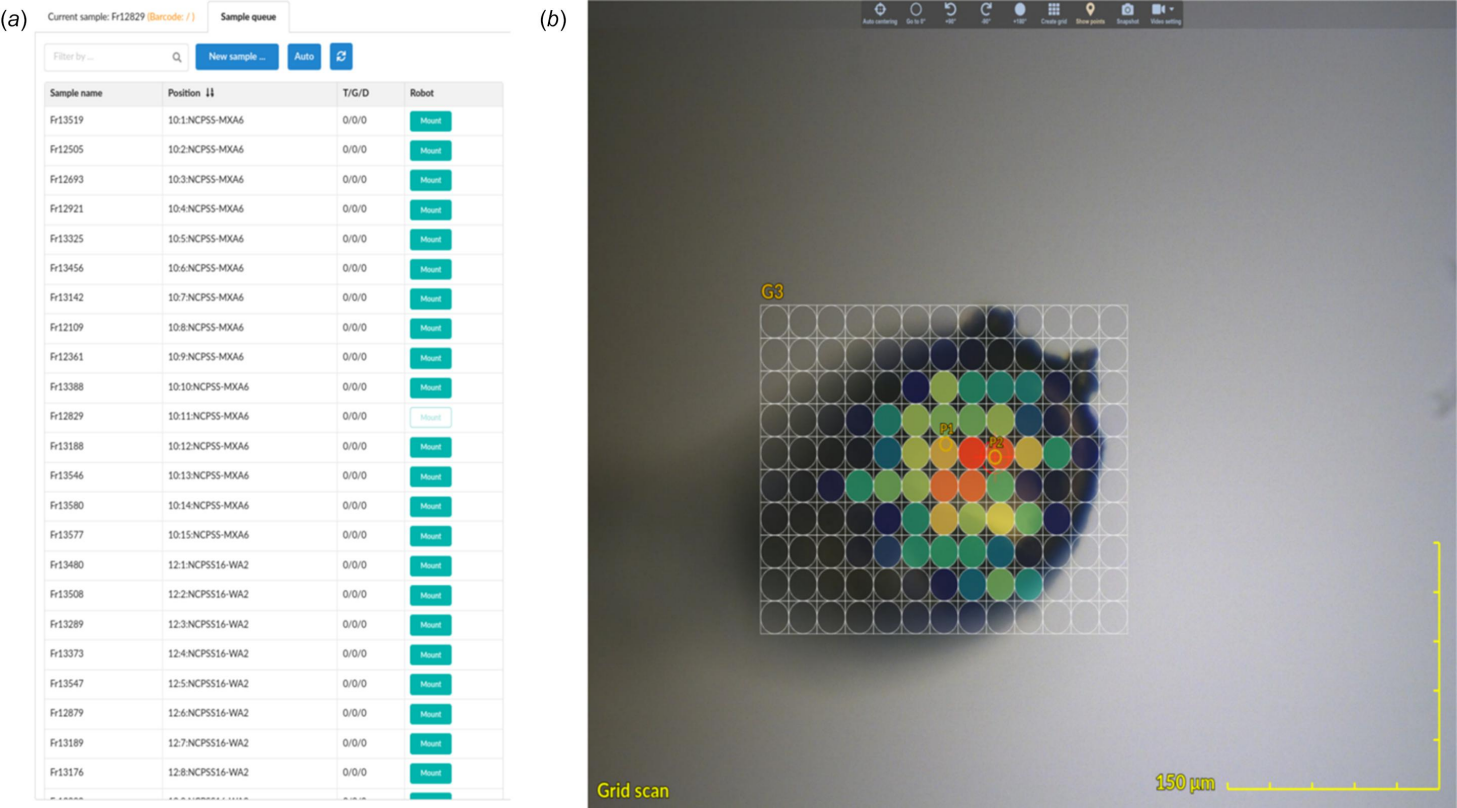
(*a*) Sample queue. Click the Mount button to the right of the specified sample name in the Sample queue column on the left to mount the corresponding sample. The sample queue can import hundreds of samples at once, which is useful for a fragment-based lead discovery (FBLD) screen campaign. (*b*) Gridscan. A heat map of the diffraction results with red corresponding to the strongest diffraction is displayed on top of the sample’s camera view. A 13 × 10 cells grid scan is covering a protein crystal. Data were collected with the Eiger S 9M detector at 40 Hz with a 20 mm × 20 mm X-ray beam. Benefiting from the stream data of the Eiger detector, the grid scan images are processed simultaneously during data collection and immediately displayed on the browser.

The left part of the interface consists of sample-related operation and information. The user can firstly import sample information and set the sample positions in the sample changer dewar using the Protocol tab, then mount or dismount the sample according to the sample name or the position in the sample changer [Fig. 3 ▸(*a*)]. In *Finback*, we employ a sample-centric operating model. All collection operation settings and their results are designed as properties of the *Sample* object. These settings and autoindexing results are also shown in this region. Autoindexing is performed asynchronously using *DIALS* (Winter *et al.*, 2018 ▸), which means that users do not need to wait for autoindexing results before starting a new data collection. This helps the experienced user maximize the efficiency of the beam time.

The middle part of the interface is the sample camera. Currently, GigE color cameras (Hikvision MV-CA013-20GC, 1/2" CMOS, 1280 × 1024 pixels) are used at SSRF BL02U1 and BL10U2. These cameras are driven by an *EPICS* Aravis driver and MJPEG video stream generated using an *FFmpeg* server. *Finback* supports MJPEG video stream directly. There are three ways to render the camera view in modern browsers: scalable vector graphics (SVG), canvas and WebGL. Among these, SVG provides the most high-level API functions and is the easiest for implementing camera views without dependending on third-party libraries. But SVG has a limit on the number of objects – too many objects will slow down the rendering speed. The initial version of *Finback* used SVG to draw the camera view, which ran smoothly in most cases. However, if more than 1000 cells were required in the grid scan, the refresh speed of the interface was too low to use. The current version of *Finback* uses *PIXI.js* to draw the camera view. *PIXI.js* is a WebGL-based 2D graphics library which can make use of the graphics card to maximize the display performance and can fall back to canvas automatically if there is no WebGL support. Over 10000 points are drawn and move smoothly with the sample movement in the grid scan [Fig. 3 ▸(*b*)]. To align the sample position, two different sample alignment methods are available: manual and automatic. In the manual option, mouse wheel clicking corresponds to sample translation and omega rotation by 90°, and mouse wheel scrolling corresponds to camera zoom. As a result, only the mouse wheel is required to align the sample, with as little as two clicks. The isolate operation mode of sample translation and rotation is also available by clicking the left mouse button. Fully automatic crystal centering is achieved using Mask-RCNN deep neural network technology (https://github.com/matterport/Mask_RCNN). This image recognition program will attempt to identify the crystal and loop at 0° and 90° before alignment. After alignment, the side of the sample with the larger area in view is rotated to align with the camera for subsequent observation and data collection.

On the right half of the interface is the motion control panel, quick crystal test collection settings panel and quick dataset collection settings panel. Routine collection tasks can be accomplished using the quick collection settings panel. If advanced collection tasks are required, such as inverse beam, helical data collection and grid scan, dialogs with detailed settings are provided. *Finback* supports inverse beam data collection using the Eiger detector. For the inverse beam, the total frame number of diffraction images can be evenly divided by the frame number of diffraction images in the sub-wedge, and .h5 files must be re-sorted after collection.

**Figure 4:**
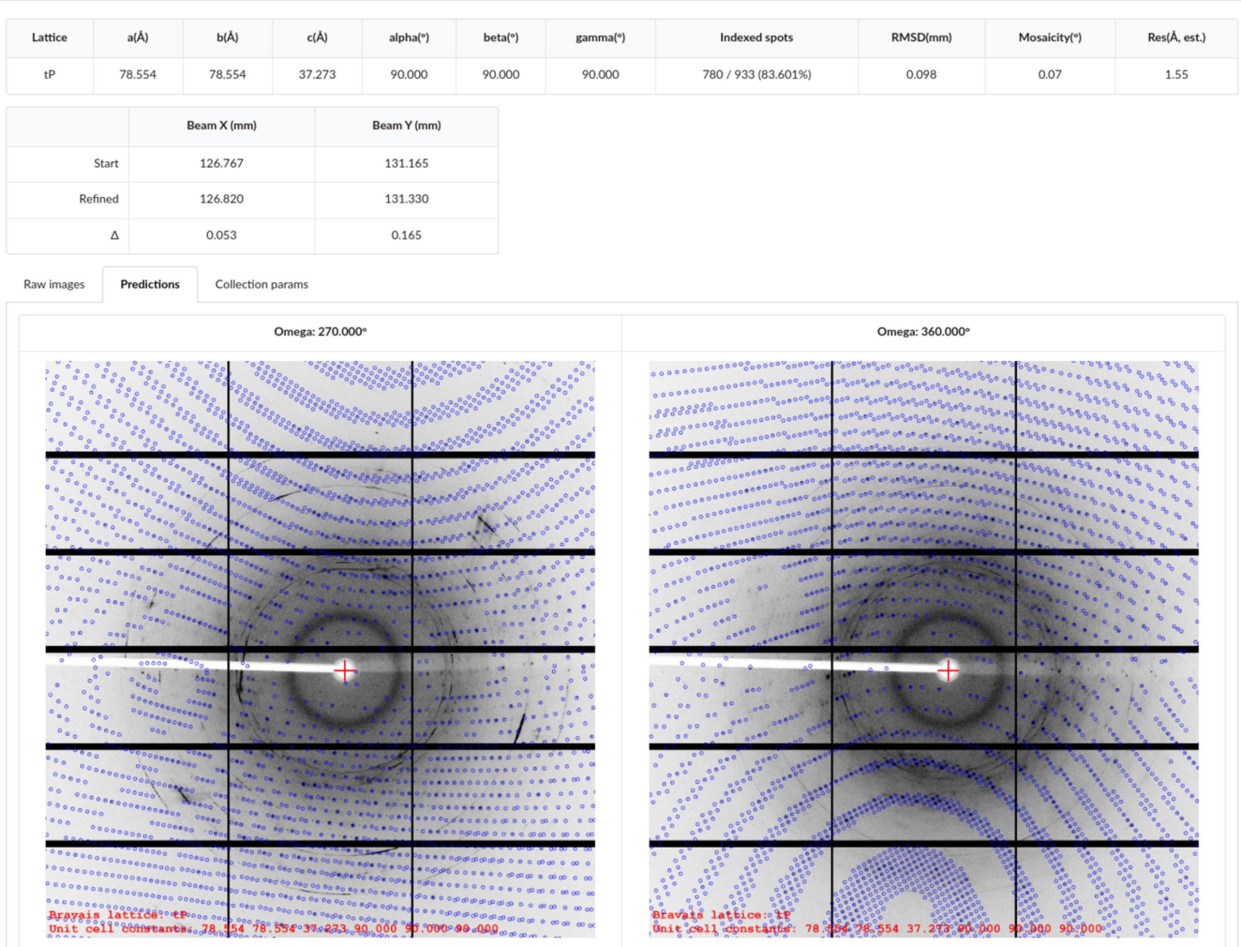
The interface for the index result and prediction. After test images are collected, *DIALS* is used to index these images, then the index result and prediction image are shown in *Finback*.

To benefit from the unified development of the software and hardware, fast multi-angle test image collection has been implemented in *Finback* . When test images need to be collected, the user can select ‘1 image’, ‘2 images every 90°’, ‘4 images every 45°’ or ‘4 images every 90°’. Whatever item is selected, only one *arm* command will be sent to the detector. When omega is rotated to the corresponding angle, the diffractometer will send 1, 2 or 4 trigger signals to the detector. In this way, the time required to collect several test images can be minimized. Furthermore, due to the lack of starting angle information for the last 1 or 3 images, these data cannot be indexed directly. To autoindex these data, *Finback* will add custom records in the master file, and a specified script to index these data, and send back the index result and prediction (Fig. 4 ▸).

### 3.2. Rawimages tab

**Figure 5:**
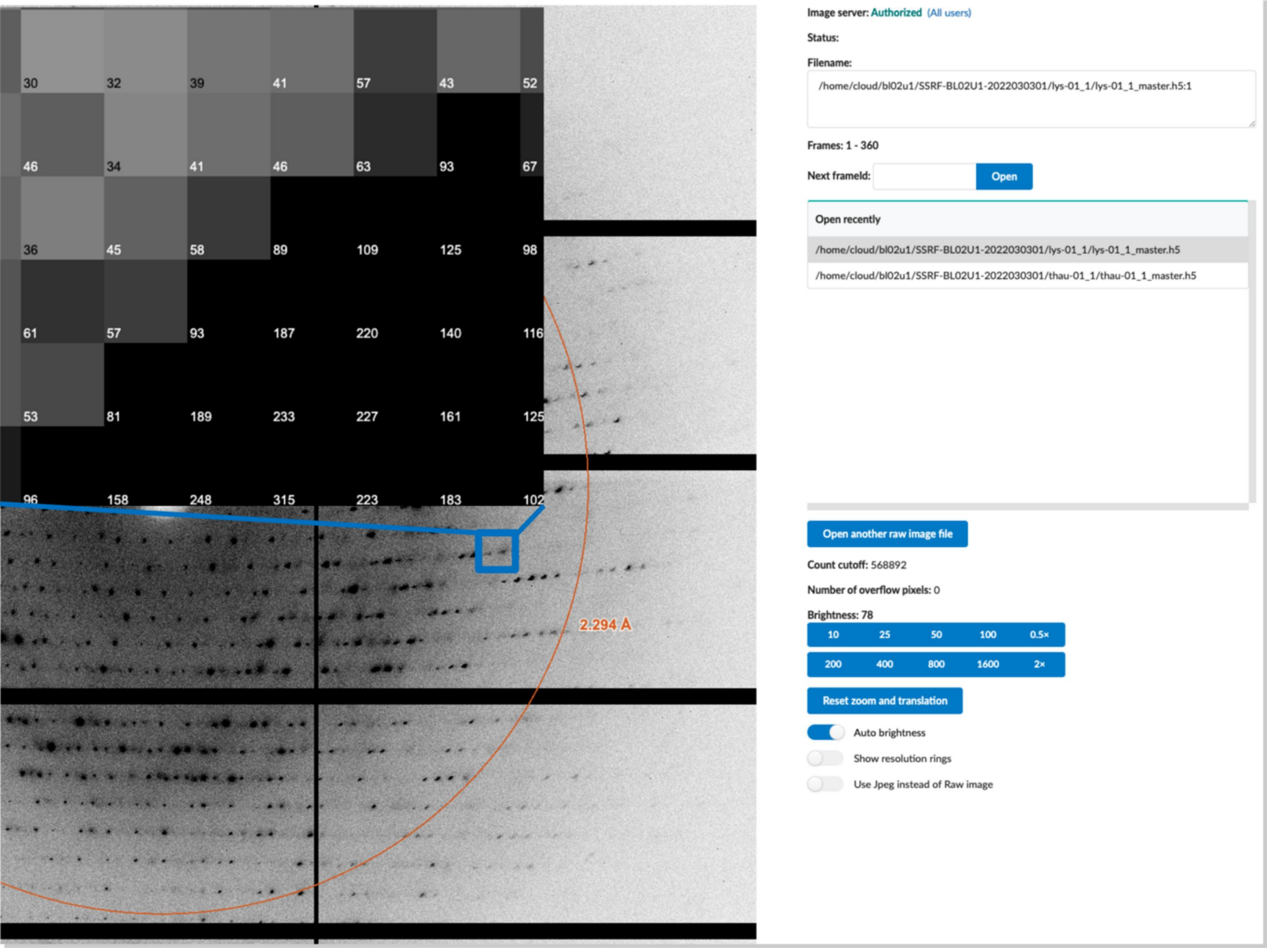
The raw image viewer of the *Finback* GUI. After test images or a dataset are collected, the first frame of the test images or the dataset will be automatically shown in the raw image viewer. For remote access, users can choose JPEG format instead of the raw diffraction images if bandwidth is too low. Besides that, any image files in the user’s home directory can be open. Users can zoom in or zoom out of images and adjust the brightness. The top-left image is local region of the raw image after zooming out. When the raw image is zoomed out at maximum magnification, the intensity of every pixel is shown.

*Finback* also supports the display of raw diffraction images in browsers for on-site and remote users (Fig. 5 ▸). Limited by network bandwidth and additional data transmission, the web raw image viewer can only display images at low frame rate and is only suitable for inspecting test images – it is not suitable for monitoring the data collection process. Depending on whether the user’s network address is outside the SSRF firewall or not, the *Finback* backend converts the raw image to zstd compression format (https://github.com/facebook/zstd) or uncompressed format, then sends these binary data via WebSocket transport protocol. For Eiger X 16M, its size is about 14 Mbytes for compression format or 70 Mbytes for uncompressed format; for Eiger2 S 9M, it is about 9 Mbytes for compression format or 35 Mbytes for uncompression format. In the *Finback* client, we also use *PIXI.js* for rendering raw images. Before rendering, the raw linearized data must be decompressed (if needed), re-shaped to a bitmap, as well as carrying out color transformation including brightness adjustment and color marking of ‘gap’, overflow pixels and bad pixels. The initial color transformation code is written in JavaScript, but only provides an acceptable performance in browsers with a high-performance just-in-time (JIT) compiler (*e.g.* Google Chrome). To be compatible with more browsers and obtain higher performance, we have re-written the color transformation code using WebAssembly technology in C language, so the raw image viewer can now change brightness and render raw images smoothly on the various modern browsers. For on-site users, we also provide a helper script called *albula_helper*. It receives commands from the *Finback* backend via ZeroRPC, then the manipulation Python API of *Albula* to display raw images in real time.

### 3.3. Multiple concurrent sessions

The *Finback* system supports multiple clients running at the same time, so several authorized users can run the *Finback* GUI at different locations together. But only one of them can operate *Finback*, which is referred to as the active client, and the others are referred to as passive clients. Any user can take control permissions from the current active user without the active user’s consent. Beamline staff can terminate remote user authorization and log them out, but staff do not have the priority to obtain control permissions over other users. This design concept is inherited from *BluIce* and is efficacious for remote access. To implement this feature, real-time hardware status information synchronization, as well as user operation information including data collection parameters, grid scan settings, collection progress and so on, must be synchronized among every client. In *Finback*, which benefits from sample-centric operating models and JavaScript object notation (JSON) data interchange format, the active client stringifies and sends the *Sample* object to all logged-in passive clients and passive clients receive and parse text information to the *Sample* object. The active client also sends a copy to the Redis database at the same time. The *Finback* client loads this copy from the Redis database on startup every time, so the latest operational information can be shown for a newly logged user or after browser refresh.

### 3.4. *Finback* configuration parameters

In order to be compatible with different beamlines, an MX data collection system needs to set many configuration parameters, such as beam center, exposure time limit, sample changer mounting positions and so on. Usually these configuration parameters are saved in files or databases that the program reads every time at startup. But if the configuration parameters are modified, the program needs to be restarted for these to take effect. *Finback* is a dedicated system for crystallography, in which the procedures are well established. Meanwhile, at the backend, there is no requirement for *Finback* to be compatible with any hardware driver other than *EPICS*. Therefore, it is feasible to directly use *EPICS* to implement the parameter setting of *Finback*. There are about 200 configuration parameters (hardware parameters not included) in *Finback*, with each configuration parameter corresponding to a PV. Some of these parameters may change irregularly, such as beam center, sample changer mounting positions, *etc*. For these parameters, the *autosave* module of *EPICS* is used to implement regular backup and restoration. Using the callback mechanism of *EPICS*, the changes of the parameters can be known to the frontend and backend of *Finback* in real time, so restarting is not necessary before the parameters take effect.

## 4. Summary

We have recently developed an MX experiment environment at SSRF, including diffractometer, sample changer, data collection system, automatic data processing pipeline and experiment information management system. As a part of this system, *Finback* is a mature and easy-to-use software system for MX data collection. It inherits some design concepts of *BluIce* that have been proven to be very effective over the years, such as server/client architecture and active/passive mechanism for remote access. In *Finback*, hardware control is unified to *EPICS*, which is more streamlined, and the maintenance work is reduced. By integrating with home-made diffractometer and sample changer, some customized features can be easily implemented. By integrating with automatic data processing and experiment information management systems, users can track samples from data collection to data processing and, finally, to inspecting and downloading automatic data processing results. The *Finback* source code can be obtained by email from the corresponding author.
